# Sarcopenia as a Marker of Immunometabolic Vulnerability in Pancreatic Ductal Adenocarcinoma

**DOI:** 10.3390/cancers18081205

**Published:** 2026-04-09

**Authors:** Mukund Karthik, Sara Shahrestani, Jin-soo Park, Christian Ratnayake, Charbel Sandroussi

**Affiliations:** 1 Sydney Medical School, The University of Sydney, Sydney, NSW 2006, Australia; sara.shahrestani@mountsinai.org (S.S.);; 2 Research, Innovation & Surgical Education (RISE), Sydney, NSW 2050, Australia; 3 Recanati/Miller Transplantation Institute, Mount Sinai Hospital, New York, NY 10029, USA; 4 Department of Upper GI Surgery, Royal Prince Alfred Hospital, Sydney, NSW 2050, Australia; 5 Department of Upper GI Surgery, Chris O’Brien Lifehouse, Sydney, NSW 2050, Australia

**Keywords:** pancreatic cancer, surgery, prehabilitation, immune dysfunction, sarcopenia, cancer cachexia

## Abstract

Despite advances in surgical technique and perioperative care, pancreatic ductal adenocarcinoma (PDAC) remains associated with poor survival. Sarcopenia is highly prevalent in PDAC and is consistently associated with inferior survival and reduced tolerance of systemic therapy. However, interventions primarily aimed at increasing muscle mass through nutritional supplementation and resistance-based exercise have yielded limited improvements in clinically meaningful postoperative outcomes. This has prompted increasing interest in sarcopenia as a marker of broader biological vulnerability rather than isolated physical deconditioning. Emerging clinical, translational, and experimental evidence demonstrates that skeletal muscle and adipose tissue function as active immunometabolic organs, and that cancer-associated inflammatory pathways drive early muscle loss, immune dysfunction, and impaired physiological recovery. Across multiple clinical cohorts, sarcopenia is reproducibly associated with worse overall survival and failure to complete adjuvant therapy, but not consistently with increased postoperative complications, suggesting that its prognostic relevance lies in impaired recovery and oncological fitness rather than immediate surgical risk. Translational studies further indicate that sarcopenia identifies patients with reduced antitumor immune competence, particularly in early-stage disease. This review synthesizes current evidence linking sarcopenia, immune dysfunction, and surgical outcomes in PDAC and examines implications for perioperative care. We propose that immunometabolic-informed prehabilitation, integrated with existing nutritional and exercise strategies, may represent a more effective approach to improving recovery, treatment tolerance, and durable oncological outcomes following PDAC resection.

## Simple Summary

Pancreatic cancer has a very poor prognosis, even when surgery is possible. Many patients with pancreatic cancer have sarcopenia, that is, low muscle mass, which is linked to poorer survival and reduced ability to complete chemotherapy. Traditional approaches to address sarcopenia have focused on nutrition and exercise to build muscle, but these strategies have shown limited benefits in improving meaningful clinical outcomes. Growing evidence suggests that loss of muscle in pancreatic cancer reflects deeper problems in immune and metabolic function rather than physical weakness alone. Muscle and fat tissue actively influence immune responses, inflammation, and recovery after surgery. This review explores how sarcopenia may identify patients with reduced biological resilience who struggle to recover and tolerate cancer treatment after surgery. We propose that future prehabilitation strategies should focus on improving immune and metabolic health, alongside nutrition and exercise, to better support recovery and long-term outcomes after pancreatic cancer surgery.

## 1. Introduction

### 1.1. Introduction to Pancreatic Adenocarcinoma

Pancreatic ductal adenocarcinoma (PDAC) prognosis is poor, with 5-year survival rates consistently below 10% despite advances in surgical technique and perioperative care [1]. Radical resection is the only potentially curative treatment. However, even among patients deemed anatomically resectable, outcomes are frequently undermined by postoperative morbidity, failure to complete adjuvant chemotherapy, and recurrence rates, which can be as high as 80% [2]. These shortcomings are poorly explained by operative factors alone and suggest that host biological vulnerability plays a critical role in determining surgical benefit [1,2,3].

### 1.2. Definition and Measurement of Sarcopenia in PDAC

Sarcopenia is defined as a reduction in skeletal muscle mass, corresponding with frailty and reduced perioperative reserve. Current studies examining the prevalence of sarcopenia in PDAC report ranges between 30–65% [2]. Currently, the most common method for measuring sarcopenia is radiological, using a single axial slice at the level of the 3rd lumbar vertebrae to quantify muscle surface area. The cutoffs for sarcopenia based on these measurements are heterogeneous in the literature [1,2] (see Table 1).

### 1.3. Clinical Impact of Sarcopenia: Survival, Treatment Tolerance, and Prehabilitation Failure

Sarcopenia has been associated with worse overall survival and disease-free survival in PDAC across numerous studies, while also being associated with intolerance to systemic adjuvant chemotherapy [1,2,3]. While malnutrition and physical deconditioning in the context of PDAC have been discussed in the literature, nutritional supplementation and resistance-based interventions have produced heterogeneous and often disappointing results [3,4,5]. A systematic review of six studies on preoperative exercise programs found that, despite improvements in muscle mass, postoperative outcomes did not differ, except for a shorter hospital stay and lower rates of delayed gastric emptying [3].

**Table 1:** Variability in radiological sarcopenia definitions across key studies in PDAC.

Study (Author/Year)	Population/Setting	Measurement Method	SMI Cutoff (Male/Female) cm^2^/m^2^	Sarcopenia Prevalence Reported
Prado et al., 2008 [6]	Obese cancer patients (mixed tumours)	L3 CT-derived SMI	52.4/38.5	N/A (derivation cohort)
Martin et al., 2013 [7]	Mixed solid tumours, Western cohorts	L3 CT-derived SMI; BMI-stratified	43–53 (BMI-stratified)/41	Variable by BMI subgroup
Choi et al., 2015 [8]	Advanced PDAC, palliative chemotherapy (Asian cohort)	L3 CT-derived SMI; ROC-derived	42.2/33.9	21.3%
Raoul et al., 2023 [1]	Pancreatic cancer (systematic review; 48 studies, *n* = 9063)	L3 CT-derived SMI	Variable: 40 to >50 range across studies	19% (<40), 45% (40–50), 57% (>50) depending on cutoff used
Thormann et al., 2023 [2]	Pancreatic cancer (meta-analysis)	L3 CT-derived SMI	Variable across included studies	45% pooled; high heterogeneity (I^2^ > 85%)
Bundred et al., 2019 [9]	Pancreatic cancer (systematic review; 42 studies, *n* = 7619)	CT (SMI) predominant; BIA and DXA also used	Multiple definitions across studies	Not pooled; wide variation noted
Kim et al., 2022 [10]	Resectable PDAC (*n* = 347)	L3 CT-derived SMI; Contal-O’Quigley method	Cohort-derived cutoffs	Not specified; AI-assisted segmentation

## 2. Reframing Sarcopenia as a Marker of Immunometabolic Vulnerability

The consistent association between sarcopenia and inferior overall survival in pancreatic ductal adenocarcinoma (PDAC) has been well established across systematic reviews and meta-analyses [1,2]. However, these same analyses demonstrate far less consistent associations between sarcopenia and short-term postoperative morbidity, pancreatic fistula rates, or length of stay [1,2]. This apparent paradox suggests that sarcopenia may not primarily reflect an impaired ability to withstand the acute physiological stress of surgery, but rather a broader host vulnerability that influences long-term oncological recovery. Traditional interpretations have framed sarcopenia as a manifestation of malnutrition or physical deconditioning. Yet prehabilitation strategies focused on exercise and nutritional loading, while capable of improving surrogate measures such as skeletal muscle index, have demonstrated limited impact on clinical outcomes in PDAC [5,9,11].

Increasingly, skeletal muscle and adipose tissue are recognized as active immunometabolic organs that regulate systemic inflammatory tone, endocrine signaling, and immune cell function [12,13]. In this context, sarcopenia may be better conceptualized not as isolated muscle depletion, but as a clinical biomarker of dysregulated host–tumour crosstalk with translational data supporting this reframing.

Myosteatosis, defined as the infiltration of adipose tissue into skeletal muscle and quantified on CT as reduced skeletal muscle density (SMD), represents a structural manifestation of this pathological crosstalk between muscle and fat. Rather than reflecting simple disuse or nutritional depletion, myosteatosis is driven by tumour-associated inflammatory signaling and intramyocellular lipid accumulation, and its presence denotes qualitative muscle deterioration distinct from loss of muscle mass alone. Two recent meta-analyses demonstrated that myosteatosis is independently associated with worse overall survival in pancreatic cancer across multiple treatment settings and patient populations [14,15]. In the resectable setting specifically, preoperative myosteatosis has been associated with inferior survival following curative-intent surgery. Additionally, in patients with metastatic PDAC, the co-existence of low muscle mass and myosteatosis independently predicted grade 3 to 4 chemotherapy toxicity and treatment-modifying events during first-line chemotherapy [10,16].

The albumin-myosteatosis gauge (AMG), defined as the product of serum albumin and SMD, attempts to capture both dimensions simultaneously by integrating muscle quality with serum albumin as a surrogate of systemic inflammatory burden and nutritional status. In a cohort of 196 patients with advanced PDAC undergoing first-line chemotherapy, lower AMG values independently predicted inferior overall survival on multivariable analysis, and the index has shown prognostic utility across other gastrointestinal malignancies [17,18]. Although prospective evaluation in the resectable perioperative setting is still needed, the AMG represents an emerging composite biomarker that aligns closely with the immunometabolic framework proposed here, capturing in a single measure the functional and inflammatory dimensions of host vulnerability that CT-defined muscle mass alone cannot fully reflect.

In patients with localized PDAC, sarcopenia has been associated with reduced tumour-infiltrating CD8^+^ T cells and inferior survival independent of tumour stage or genomic, suggesting that host body composition may reflect the functional state of antitumor immunity [19]. Similarly, altered cytokine profiles, including reduced hepatic IL-4 expression in cachectic patients, indicate qualitative immune signaling disruption rather than simply elevated systemic inflammation [20].

Preclinical models further demonstrate that muscle wasting and systemic inflammatory activation occur early in pancreatic tumorigenesis, even before invasive disease develops [21]. Together, these observations support the concept that sarcopenia in PDAC reflects an immunometabolic phenotype characterized by chronic inflammatory signaling, altered substrate metabolism, and impaired immune resilience. For the surgeon, this reframing shifts the clinical question from “Can this patient survive the operation?” to “Can this patient recover sufficiently to complete multimodal therapy and sustain antitumor immunity?”

## 3. Sarcopenia, Cancer Cachexia and Immune Dysfunction in PDAC: Mechanistic Insights

A mechanistic interrogation of cancer cachexia in PDAC reveals several interconnected signaling axes that link tumour biology, systemic inflammation, skeletal muscle atrophy, and immune dysfunction. These pathways provide biological plausibility for viewing sarcopenia as a manifestation of host immunometabolic dysregulation rather than simply muscle wasting from poor nutrition and reduced use (see Table 2).

### 3.1. IL-6/STAT3 Axis Signaling and Systemic Inflammatory Crosstalk

Interleukin-6 (IL-6) is one of the most extensively characterized mediators of pancreatic cancer cachexia. Early clinical observations demonstrated associations between elevated cytokines, acute-phase responses, and increased resting energy expenditure in patients with pancreatic cancer [22]. Subsequent mechanistic work has shown that tumour-derived IL-6, including trans-signaling between tumour, adipose tissue, and muscle, contributes directly to muscle wasting [23].

Hepatic STAT3 activation has also been shown to drive early-stage pancreatic cancer cachexia through suppression of ketogenesis and systemic metabolic reprogramming [24]. This highlights the liver as a central mediator of tumour-induced metabolic stress rather than muscle acting in isolation.

Importantly, translational attempts to interrupt this pathway have entered clinical testing. In a randomized phase II study combining nab-paclitaxel/gemcitabine with the IL-6 receptor inhibitor tocilizumab in advanced, unresectable PDAC, investigators explored survival and cachexia-related outcomes, finding that patients that received tocilizumab had significantly less muscle loss. However, survival or oncologic benefit was not demonstrated, and tocilizumab recipients had a higher rate of adverse events [25]. Although not conducted in the resectable or neoadjuvant setting, such studies provide proof of concept that inflammatory cytokine blockade, when incorporated into treatment pathways, may modulate systemic catabolism in pancreatic cancer.

Collectively, the IL-6/STAT3 axis integrates tumour burden, hepatic metabolism, systemic inflammation, and skeletal muscle atrophy, reinforcing the concept of sarcopenia as a multi-organ immunometabolic syndrome.

### 3.2. Activin-Myostatin Signaling and Muscle Growth Suppression

The activin–myostatin pathway represents a second major axis implicated in PDAC-associated muscle wasting. Preclinical inhibition of activin receptor type IIB (ACVR2B) signaling was shown to attenuate muscle wasting and improve survival in pancreatic cancer murine models [26]. Elevated circulating activins have been identified in pancreatic cancer, suggesting a systemic response with implications for targeted therapy [27].

Clinical translation has been attempted with anti-myostatin antibodies. In a phase II trial of LY2495655 in pancreatic cancer, inhibition of myostatin signaling was evaluated for effects on muscle mass and physical function [28]. Although functional improvements were modest, survival benefits were not demonstrated. However, this trial found that those who were pre-cachectic or only had modest muscle loss to begin with had better survival outcomes, indicating that targeting this axis may have therapeutic benefit before a patient is truly sarcopenic, as opposed to reversing the condition. Regardless, this trial represents one of the few examples of direct cachexia-targeted therapy in PDAC.

Importantly, activin/myostatin signaling intersects with broader inflammatory and TGF-β pathways, suggesting that muscle growth suppression in PDAC is an actively maintained molecular state [29,30]. In surgical oncology, these studies demonstrate that upstream regulators of muscle homeostasis are pharmacologically modifiable, although optimal timing and patient selection remain undefined.

### 3.3. TGF-β/SMAD Signaling and Fibrotic Muscle Remodeling

Transforming growth factor-β (TGF-β), the expression of which is encoded by SMAD3, is highly relevant to cancer-induced cachexia, sarcopenia, and fibrosis. Experimental models demonstrate that TGF-β signaling induces skeletal muscle atrophy and fibrosis through upregulation of atrogin-1 and scleraxis [31]. SMAD3 activation further inhibits mechanistic target of rapamycin (mTOR) signaling and protein synthesis, promoting muscle wasting in vivo [32].

In pancreatic cancer specifically, TGF-β, along with Kruppel-like factor 10 (KLF10) signaling, has been shown to regulate atrophy-associated genes and induce muscle wasting [33]. Reviews of TGF-β signaling in cancer-induced cachexia emphasize its dual role in fibrosis and immune modulation, while targeting this pathway in murine pancreatic cancer models reduced muscle loss [34]. TGF-β is also a known suppressor of cytotoxic T-cell activity within the tumour microenvironment, providing a mechanistic bridge between muscle wasting and impaired antitumor immunity. A key difficulty in targeting the KLF10 pathway is that, although it is associated with sarcopenia and muscle loss in pancreatic cancer, it has been shown to act as a tumour suppressor in other systems [33].

### 3.4. Mitochondrial Dysfunction and Energetic Failure

Beyond inflammatory signaling, mitochondrial dysfunction has emerged as a central component of cancer cachexia. Integrative studies in murine pancreatic cancer models demonstrate impaired oxidative phosphorylation and altered mitochondrial architecture in skeletal muscle [35]. These abnormalities contribute to reduced energetic efficiency and fatigue independent of absolute muscle mass.

Mitochondrial dysfunction has been implicated as a contributor to impaired skeletal muscle quality, particularly in ageing muscle, where alterations in mitochondrial morphology and accumulation of reactive oxygen species promote myocyte apoptosis and reduced oxidative capacity [12,35]. In PDAC-associated cachexia, these findings suggest that CT-defined muscle loss may represent only one dimension of a broader energetic impairment. Radiologic sarcopenia remains a reproducible prognostic marker, but it may incompletely capture qualitative deficits in muscle bioenergetics that influence physiological reserve [1,2,35].

Preclinical studies in ageing-related sarcopenia have proposed modulating intracellular mitochondrial substrates and redox pathways as a potential strategy to restore oxidative capacity [36]. However, these concepts have not been evaluated in cancer-associated sarcopenia and remain speculative in the context of PDAC. At present, mitochondrial dysfunction in resectable pancreatic cancer should therefore be viewed as a hypothesis-generating mechanistic framework that may help explain heterogeneity in treatment tolerance and postoperative recovery, rather than a validated perioperative therapeutic target.

### 3.5. Linking Mechanism to Clinical Phenotype

These interconnected pathways: IL-6/STAT3, activin–myostatin, TGF-β/SMAD, and mitochondrial dysfunction collectively describe a state of sustained inflammatory signaling, suppressed anabolic pathways, fibrotic remodeling, and impaired cellular energetics. Importantly, many of these axes also regulate immune cell differentiation and the composition of the tumour immune microenvironment [13].

This convergence provides a biological framework for the clinical observation that sarcopenia is strongly associated with inferior survival and reduced tolerance of systemic therapy, yet inconsistently associated with immediate postoperative morbidity [1,2]. Rather than identifying patients at risk of surgical catastrophe, sarcopenia may identify those at risk of immunometabolic failure during the critical transition from surgery to adjuvant therapy.

In surgical trial design, these mechanistic insights justify exploring multimodal strategies that integrate nutritional optimization, exercise, and targeted modulation of upstream inflammatory and sarcopenia-associated pathways [29,30,37]. Whether such approaches can meaningfully alter long-term oncological outcomes in resectable PDAC remains to be determined, but the biological rationale is increasingly compelling.

**Table 2:** Key studies on sarcopenia and immunometabolic dysfunction in PDAC.

Pathway	Key Study (Author/Year)	Model/Setting	Effect on Skeletal Muscle	Effect on Immune/Systemic Biology	Translational or Therapeutic Implication
IL-6/STAT3 signaling	Falconer, 1994 [22]	Human pancreatic cancer cohort	Associated with increased resting energy expenditure	Elevated cytokines; acute-phase response	Early clinical evidence of systemic inflammatory metabolism
	Rupert, 2021 [23]	Human + murine PDAC models	Tumour-derived IL-6 drives muscle wasting via trans-signaling	Crosstalk between tumour, fat, and muscle	Demonstrates multi-organ inflammatory axis
	Arneson-Wissink, 2024 [24]	Murine PDAC	Hepatic STAT3 suppresses ketogenesis; systemic metabolic reprogramming	Identifies liver as mediator of cachexia	Expands cachexia beyond muscle-centric model
	Chen, 2025 [25]	Phase II trial (advanced PDAC)	Evaluated cachexia-related outcomes	IL-6 receptor blockade feasible in humans	Investigating utility of pathway modifiability
Activin–Myostatin signaling	Zhong, 2019 [27]	Human + experimental	Elevated activins suppress muscle growth	Endocrine dysregulation	Identifies systemic activin response
	Nissinen, 2018 [26]	Murine cancer model	ACVR2B blockade attenuates wasting; improves survival	Alters mTOR localization	Demonstrates survival signal in preclinical model
	Golan, 2018 [28]	Phase II trial (pancreatic cancer)	Myostatin inhibition evaluated for muscle function	Modest functional benefit	Demonstrates feasibility of direct anti-cachexia targeting
TGF-β/SMAD signaling	Mendias, 2012 [31]	Experimental	Induces atrogin-1; promotes atrophy and fibrosis	Fibrotic remodeling	Mechanistic basis of muscle remodeling
	Goodman, 2013 [32]	Experimental	SMAD3 inhibits mTOR and protein synthesis	Promotes catabolic transcriptional programs	Links signaling to anabolic suppression
	Dasgupta, 2023 [33]	Murine PDAC	TGF-β/KLF10 axis induces atrophy-associated genes	Intersects with tumour signaling	PDAC-specific atrophy pathway
	Balsano, 2022 [34]	Review (cancer cachexia)	Central role in cancer-induced muscle wasting	TGF-β suppresses cytotoxic T-cell activity	Dual muscle–immune axis
Mitochondrial dysfunction	Gicquel, 2024 [35]	Murine PDAC	Impaired oxidative phosphorylation; altered mitochondrial architecture	Energetic inefficiency	Suggests qualitative muscle dysfunction
	Poulia, 2020 [12]	Review (PDAC cachexia)	Metabolic dysregulation in muscle	Systemic inflammatory–metabolic shift	Supports immunometabolic framing
Host–Tumour Immune Interface	Masuda, 2023 [19]	Human resectable PDAC	CT-defined sarcopenia	Reduced tumour-infiltrating CD8^+^ T cells; worse survival	Links muscle phenotype to antitumor immunity
	Prokopchuk, 2017 [20]	Human translational	Cachexia-associated	Reduced IL-4 signaling	Qualitative immune alteration in cachexia
	Wiktorin, 2024 [38]	Human PDAC (perioperative)	N/A	Surgery-induced Myeloid Derived Suppressor Cell expansion associated with survival	Highlights perioperative immune suppression

## 4. Sarcopenia and the Tumour Microenvironment

The relationship between systemic muscle wasting and the tumour microenvironment in PDAC is unclear but is increasingly recognized as bidirectional. Sarcopenia has been associated with reduced tumour-infiltrating CD8^+^ T-cell density and inferior survival in resectable PDAC, suggesting that body composition may reflect host immune competence within the tumour bed [19].

Mechanistic pathways implicated in cancer cachexia substantially overlap with those implicated in tumour immune regulation. IL-6/STAT3 signaling promotes both muscle atrophy and immune cell exhaustion [23,24]. TGF-β signaling contributes to skeletal muscle fibrosis while simultaneously suppressing cytotoxic T-cell activity within the tumour microenvironment [33,34]. Nuclear Factor Kappa B (NF-κB) activation drives proteolysis and inflammatory amplification, shaping both systemic cytokine profiles and immune cell differentiation [30,39].

Perioperatively, surgical stress further alters immune equilibrium. Expansion of myeloid-derived suppressor cells and activation of reactive oxygen species pathways have been associated with postoperative survival in PDAC [38]. In patients already characterized by immunometabolic vulnerability, additional perioperative immune suppression may impair antitumor surveillance during a critical window following resection, thereby affecting the survival trajectory.

Together, these data support a conceptual model in which sarcopenia reflects systemic immune dysregulation that is mirrored within the tumour microenvironment. CT-defined muscle loss may therefore serve as a surrogate for impaired host–tumour immune interactions, providing a visible biomarker of a deeper immunological phenotype.

## 5. Implications for Surgical Prehabilitation in PDAC

The mechanistic pathways described above suggest that sarcopenia in PDAC represents a systemic inflammatory–metabolic phenotype rather than isolated muscle depletion. This distinction has important implications for the design of prehabilitation strategies. Traditional prehabilitation in hepatopancreatobiliary surgery has centered on resistance exercise, protein supplementation, and optimization of nutritional status [5,9]. While these interventions can improve skeletal muscle index or aerobic capacity, improvements in clinically meaningful outcomes, particularly chemotherapy completion and survival, remain inconsistent [5,11] (see Table 3).

The limited impact of muscle-centric interventions may reflect a mismatch between therapeutic target and underlying biology. In PDAC, muscle wasting is actively driven by tumour-associated cytokine signaling, TGF-β–mediated transcriptional reprogramming, NF-κB activation, and mitochondrial dysfunction [30,33,35]. Simply increasing caloric intake or prescribing resistance training does not directly interrupt these upstream drivers. Moreover, the preoperative window in PDAC is often short—typically 4–6 weeks in upfront resection and variable during neoadjuvant therapy—limiting the feasibility of substantial hypertrophic gains [5].

**Figure 1:**
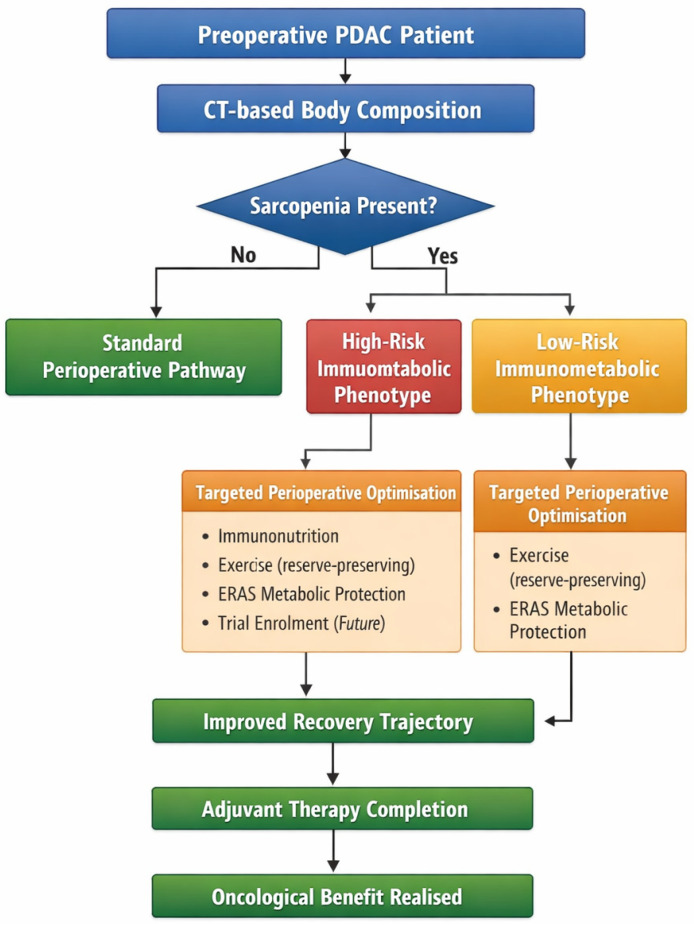
This schematic outlines a proposed approach to perioperative management in patients with pancreatic ductal adenocarcinoma based on the premise that sarcopenia represents a marker of underlying immunometabolic vulnerability rather than isolated muscle depletion. Preoperative CT-based body composition assessment is used to identify sarcopenia and stratify patients into immunometabolic risk phenotypes. Patients without sarcopenia proceed through a standard perioperative pathway, whereas those with sarcopenia undergo targeted perioperative optimization tailored to immunometabolic risk severity. Interventions may include immunonutrition, reserve-preserving exercise, enhanced recovery metabolic strategies, and potential enrolment in future immunometabolic trials. The primary objective is not merely a reduction in immediate surgical morbidity, but improvement in postoperative recovery trajectory, enabling completion of adjuvant therapy and realization of oncological benefit.

An immunometabolic framework reframes the primary objective of prehabilitation. Rather than aiming to reverse sarcopenia, the goal becomes attenuation of systemic inflammatory signaling and supporting perioperative immune competence to get patients from surgery to successful completion of adjuvant therapy, depending on whether they are sarcopenic and if they are of a high-risk immunometabolic phenotype (Figure 1).

### 5.1. Immunonutrition and Inflammatory Modulation

Immunonutrition protocols incorporating arginine, omega-3 fatty acids, and nucleotides have been proposed to attenuate perioperative inflammatory stress and support immune competence in gastrointestinal oncology [40]. In elderly patients undergoing pancreaticoduodenectomy, composite immunonutritional indices, such as the neutrophil–lymphocyte ratio and the prognostic nutritional index, correlate with postoperative survival [4]. These associations suggest immunonutrition protocols that may be measured and evaluated by these indices may aid in targeting underlying pathways linked to sarcopenia in PDAC.

Mechanistically, omega-3 fatty acids alter eicosanoid synthesis and may reduce pro-inflammatory cytokine amplification, while arginine supports T-cell proliferation and nitric oxide–mediated immune signaling [40]. Unlike pharmacologic cytokine blockade, immunonutrition does not directly inhibit upstream sarcopenia pathways, but it may act by modulating downstream inflammatory balance during the perioperative period, when surgical stress superimposes on tumour-driven muscle catabolism.

Within an immunometabolic framework, the relevance of immunonutrition may not lie in reversing established sarcopenia, but in potentially attenuating the additive perioperative inflammatory burden. Whether such modulation meaningfully improves chemotherapy tolerance or long-term oncologic outcomes in PDAC remains uncertain and warrants prospective evaluation.

### 5.2. Exercise as an Immunometabolic Intervention

Exercise-based prehabilitation is frequently justified on the basis of muscle hypertrophy; however, other relevant perioperative effects may be immunometabolic. Skeletal muscle contraction induces the release of myokines that modulate innate and adaptive immunity, improve insulin sensitivity, and attenuate systemic inflammation [13]. In PDAC, home-based exercise during preoperative treatment has been associated with preservation of skeletal muscle index, although gains in muscle density were limited and the study was not powered to detect differences in postoperative or oncological outcomes [11].

Accordingly, while exercise may help maintain physiological reserve during neoadjuvant therapy, evidence that modest skeletal muscle preservation translates into improved survival or reduced postoperative morbidity remains limited. Given emerging data on mitochondrial dysfunction in pancreatic cancer–associated muscle wasting, aerobic conditioning may theoretically enhance oxidative capacity and metabolic flexibility; however, this remains theoretical rather than clinically established [35]. At present, exercise should be viewed as a strategy to mitigate functional decline during systemic therapy rather than as a proven method of reversing sarcopenia or altering oncological outcomes.

### 5.3. ERAS and Metabolic Protection

Enhanced Recovery After Surgery (ERAS) protocols aim to minimize perioperative metabolic stress by carbohydrate loading, avoiding prolonged fasting, initiating early enteral nutrition, and attenuating catabolic responses [40]. These measures may indirectly support immune recovery by limiting surgical stress–induced inflammatory cascades.

Perioperative immune suppression is increasingly recognized in pancreatic cancer. Surgery-induced myeloid-derived suppressor cell (MDSC) expansion and activation of the NADPH oxidase 2 (NOX2)/reactive oxygen species (ROS) axis have been associated with postoperative survival in human PDAC [38]. If sarcopenia reflects pre-existing immune dysregulation, attenuating additional perioperative immune suppression becomes particularly relevant. ERAS principles, when integrated within an immunometabolic framework, may help mitigate additive stress responses in vulnerable patients.

### 5.4. Beyond Supportive Care: Targeted Pathway Modulation

While prehabilitation currently emphasizes supportive interventions, several malignancies have progressed toward direct modulation of sarcopenia-associated pathways. As described before, activin/myostatin inhibition has been evaluated in pancreatic cancer and in preclinical models, which improves muscle mass and survival [26,27,28]. Similarly, TGF-β–mediated atrophic signaling has been characterized as a therapeutic target albeit with limitations due to its protective effects in other cancers [33,34].

Although these agents have not been evaluated in the perioperative setting for resectable PDAC, their existence underscores an important principle—muscle wasting is not merely a passive phenomenon but a therapeutically targetable biological process. Future integration of pharmacologic sarcopenia modulation into perioperative pathways may extend prehabilitation beyond lifestyle and simple immunonutrition modifications.

Taken together, these considerations support a shift from viewing the prehabilitation of sarcopenia in PDAC as restoring muscle mass to improve surgical fitness, but toward immunometabolic optimization for surgery and subsequent oncological care. The clinically relevant endpoint should prioritize sustained recovery sufficient to enable adjuvant therapy and durable immune surveillance as opposed to just immediate and long-term postoperative survival.

**Table 3:** Summary of key prehabilitation interventions in PDAC.

Study (Author/Year)	Study Design/Setting	Intervention	Primary Endpoint/Outcome Measured	Key Finding	Immunometabolic Relevance
Parker et al., 2021 [11]	Non-randomised two-arm study; preoperative PDAC (*n* = 97)	Home-based aerobic and resistance exercise during neoadjuvant treatment	Change in SMI and SMD between treatment planning and restaging CT	Exercise group maintained SMI (0.2 ± 3.2 cm^2^/m^2^); usual care group lost SMI (−1.4 ± 3.8 cm^2^/m^2^; *p* = 0.03). No significant SMD difference between groups	Demonstrates exercise can attenuate muscle mass loss during neoadjuvant therapy, but muscle quality (SMD) unaffected. Consistent with upstream biological drivers of myosteatosis
Bundred et al., 2019 [9]	Systematic review and meta-analysis; pancreatic cancer (42 studies, *n* = 7619)	Body composition assessment; exercise and nutritional prehabilitation were reported	Overall survival, postoperative complications, perioperative mortality	Sarcopenia associated with perioperative mortality (OR 2.40) and reduced OS; not significantly associated with complications or fistula. Prehabilitation evidence limited and heterogeneous	Highlights the paradox driving this review: sarcopenia predicts survival but not short-term morbidity, supporting immunometabolic rather than surgical risk framing
De Luca et al., 2023 [40]	Narrative review; upfront resectable and borderline resectable PDAC	Immunonutrition (arginine, omega-3, nucleotides), ERAS protocols, prehabilitation during neoadjuvant treatment	Postoperative infections, length of stay, nutritional status, oncological outcomes	Preoperative immunonutrition supported by ERAS guidelines to reduce infections and length of stay. Neoadjuvant window identified as optimal for multimodal nutritional intervention. Shift from rehabilitation to prehabilitation approach recommended	Most directly relevant to clinical translation. Explicitly addresses immunonutrition in PDAC in the neoadjuvant era and recommends proactive immunometabolic intervention
Christopher et al., 2023 [5]	Narrative review; HPB cancers including PDAC	Multimodal exercise and nutrition prehabilitation	Postoperative complications, body composition, functional capacity, nutritional biomarkers	Evidence supports integration of exercise and nutrition prehabilitation in HPB cancers, improvements in surrogate endpoints but limited data on survival and chemotherapy completion. Optimal timing and components remain undefined	Reinforces that current prehabilitation targets surrogate endpoints rather than oncological outcomes. Supports argument for immunometabolic endpoint redesign
Tsukagoshi et al., 2024 [3]	Narrative review; PDAC	Nutritional supplementation, exercise, and resistance-based interventions	Survival, chemotherapy tolerance, postoperative outcomes	Heterogeneous and often disappointing results from nutritional and exercise interventions; prehabilitation does not consistently improve meaningful clinical outcomes	Provides direct support for the argument that muscle-centric prehabilitation is insufficient and an immunometabolic reframing is needed

## 6. Future Directions, Trial Design and Clinical Translation

### 6.1. Perioperative Trial Design and Research Priorities

If sarcopenia in PDAC reflects immunometabolic vulnerability, perioperative research must evolve accordingly. Current prehabilitation trials often prioritize short-term outcomes, such as length of stay, complication rates, and changes in muscle index [5,9]. However, observational data consistently demonstrate that sarcopenia is more strongly associated with overall survival and chemotherapy intolerance than with immediate surgical complications [1,2].

This discrepancy suggests that future perioperative trials in PDAC should prioritize endpoints aligned with immunometabolic resilience, including completion of adjuvant chemotherapy, time to adjuvant therapy initiation, maintenance of dose intensity, immune recovery markers (e.g., lymphocyte subsets, inflammatory cytokines), and recurrence-free survival.

Sarcopenia is currently defined predominantly radiologically using L3 CT-derived skeletal muscle indices [1,2]. While practical and simple, this approach does not distinguish between patients with isolated muscle loss and those with profound systemic inflammatory activation, making it difficult to stratify patients that may be at higher risk, or patients that may benefit from immunonutrition prehabilitation protocols. Integration of inflammatory biomarkers such as IL-6 levels, metabolic indices, or tumour immune infiltration metrics may improve sarcopenia phenotypic stratification in PDAC.

In upfront resection, the preoperative window is often limited. In neoadjuvant paradigms, however, patients may receive systemic therapy for several months prior to surgery. This extended window may provide a more feasible timeframe for multimodal immunometabolic intervention.

Exercise during neoadjuvant therapy has demonstrated preservation of muscle mass and prehabilitation prior to chemotherapy across solid tumours has been associated with improved treatment tolerance [11,41]. Embedding immunometabolic optimization within neoadjuvant treatment protocols may therefore represent a particularly promising strategy in PDAC and the timing of these interventions should be considered in the design of future trials targeting sarcopenia in PDAC.

As systemic therapies evolve, the perioperative immunometabolic state may influence treatment response. IL-6 blockade has already been evaluated alongside chemotherapy in advanced pancreatic cancer [25]. Although not designed for the resectable setting, such trials demonstrate the feasibility of combining inflammatory pathway modulation with cytotoxic regimens.

Similarly, targeting activin–myostatin signaling or TGF-β–mediated fibrosis may have downstream effects on tumour microenvironment and immune surveillance [27,28,33]. The interplay between host immunometabolism and systemic therapy response warrants dedicated investigation, as successful completion of adjuvant therapy is a fundamental objective in treating sarcopenic phenotypes in the prehabilitation setting.

Future randomized trials in resectable PDAC should therefore evaluate multimodal prehabilitation strategies that combine exercise-based metabolic conditioning, immunonutritional support, ERAS-driven metabolic protection, and biomarker-guided inflammatory modulation. Such trials should be powered not solely for short-term morbidity reduction, but for meaningful oncological endpoints, including recurrence-free survival and chemotherapy completion.

### 6.2. Clinical Implications of the Immunometabolic Framework

The mechanistic and clinical evidence synthesised in this review collectively identifies a subgroup of PDAC patients whose sarcopenia reflects not isolated muscle depletion but a broader state of immunometabolic vulnerability, characterised by chronic inflammatory signalling, suppressed anabolic pathways, and impaired perioperative immune competence. Translating this reframing into clinical practice requires a shift in the questions being asked across three domains: surgical decision-making, neoadjuvant therapy, and chemotherapy tolerance.

In surgical decision-making, the conventional role of body composition assessment has been to estimate operative risk. Within an immunometabolic framework, the clinical question shifts from whether a patient can survive the operation to whether a patient can recover sufficiently to complete multimodal therapy and sustain antitumour immunity following resection [1,2,19]. Preoperative CT-based body composition assessment, already routinely performed in PDAC staging, offers an opportunity to identify patients with an adverse immunometabolic phenotype before any treatment decision is made. For sarcopenic patients, this should prompt earlier multidisciplinary discussion about the timing and sequencing of treatment, the feasibility and goals of prehabilitation, and realistic expectations around adjuvant therapy completion rather than focusing solely on immediate postoperative morbidity.

In the context of neoadjuvant therapy, the extended preoperative window, typically several months in patients receiving systemic treatment before surgery, represents the most clinically feasible opportunity for immunometabolic intervention in PDAC [11,40,41]. In other gastrointestinal malignancies, sarcopenia developing during neoadjuvant therapy has been associated with substantially worse overall survival and recurrence-free survival, and significant muscle loss occurs in a large proportion of patients during treatment [42]. This experience from other cancers suggests that in PDAC, monitoring body composition and inflammatory biomarkers longitudinally during neoadjuvant therapy, rather than only at diagnosis, may better capture the dynamic nature of immunometabolic deterioration. The goal of intervention during this window should not be framed solely as building muscle mass before surgery, but as attenuating the inflammatory and catabolic signalling that drives immunometabolic vulnerability and ultimately impairs recovery and oncological fitness [5,40].

With respect to chemotherapy tolerance, the immunometabolic phenotype has direct implications for treatment planning that extend beyond general nutritional optimisation. Evidence from PDAC cohorts demonstrates that the combination of low muscle mass and myosteatosis, reflecting both quantitative and qualitative muscle deterioration, independently predicts grade 3 to 4 chemotherapy toxicity and treatment-modifying events during first-line chemotherapy [16]. Routine pretreatment body composition assessment could therefore inform decisions around chemotherapy regimen intensity, monitoring frequency, and the proactive integration of immunonutritional support alongside systemic therapy, rather than introducing these measures only after toxicity has occurred [4,40]. While prospective evidence supporting body composition guided chemotherapy dosing in PDAC remains limited, the biological rationale is increasingly coherent, and this represents a priority area for future interventional research.

## 7. Limitations of Current Evidence

Despite accumulating mechanistic and clinical data, important limitations temper the interpretation of the current literature. First, definitions of sarcopenia remain heterogeneous. Studies employ variable L3 skeletal muscle index cut-offs derived from sex-specific and population-specific cohorts, limiting cross-study comparability [1,2] (see Table 1). Few studies integrate muscle quality, inflammatory biomarkers, or functional measures into a composite phenotype that can be used to risk-stratify sarcopenic patients based on immunometabolic characteristics.

This variability extends beyond simple numerical thresholds and reflects deeper methodological inconsistencies that meaningfully affect the interpretation of the existing literature. Cutoff values for skeletal muscle index derived from Western, Asian, and mixed cohorts differ substantially, and their application across populations introduces systematic misclassification that inflates heterogeneity in pooled analyses [2,3]. Muscle quality assessment presents a further challenge. Skeletal muscle radiodensity, the principal CT-based surrogate of myosteatosis, lacks a universally accepted threshold, with studies variously applying fixed Hounsfield unit cutoffs or BMI-stratified definitions, meaning that the prevalence and prognostic impact of myosteatosis varies considerably depending on the criteria used [1]. Beyond measurement, few studies in PDAC move beyond a binary sarcopenic or non-sarcopenic classification to account for the spectrum of body composition phenotypes, including concurrent myosteatosis, visceral adiposity, or sarcopenic obesity, each of which may carry distinct biological and prognostic implications [3]. Until consensus definitions are established and validated across diverse PDAC populations, comparison across studies will remain limited, and the translation of body composition findings into clinical decision-making will be constrained.

Second, most clinical evidence linking sarcopenia to outcomes in PDAC derives from retrospective observational cohorts. Confounding by tumour stage, treatment selection bias, and reverse causality, whereby more aggressive disease drives muscle loss, cannot be fully excluded [3]. Prospective, biomarker-integrated studies remain limited.

Thirdly, interventional evidence is sparse. Exercise and nutritional prehabilitation trials in PDAC are small, heterogeneous, and underpowered for long-term oncologic endpoints [5,9]. Pharmacologic targeting of sarcopenia-associated pathways has largely been confined to advanced disease settings, and translation into the perioperative resectable context remains unexplored [25,28].

Finally, mechanistic data are predominantly derived from murine models. While these provide biological plausibility, extrapolation to human perioperative immunobiology requires caution [24,33]. Clarifying the causal relationship between pathway modulation and oncologic outcomes is a key future research priority.

## 8. Reframing Surgical Success in PDAC

The cumulative clinical and mechanistic data reviewed here suggest that sarcopenia in PDAC is unlikely to represent a purely structural deficit of skeletal muscle. Rather, it appears to reflect a broader immunometabolic state characterized by tumour-driven inflammatory signaling, altered systemic metabolism, and impaired host immune competence [12,23,33]. This distinction is clinically meaningful. While sarcopenia is consistently associated with inferior overall survival and reduced tolerance of systemic therapy, its association with immediate postoperative complications remains inconsistent [1,2,3]. Such findings suggest that sarcopenia may be less a predictor of operative fragility and more a marker of diminished physiological and immunological resilience during the perioperative and adjuvant phases of care.

In resectable PDAC, the association between sarcopenia and reduced tumour-infiltrating CD8^+^ T-cell density, as well as the IL-6/STAT3 and TGF-β/SMAD signaling axes that drive both muscle catabolism and immune modulation, strengthens the interpretation that the muscle phenotype observed on CT may serve as a visible surrogate for underlying immunological dysfunction [19,24,33,34].

This reframing has important implications for perioperative strategy. Traditional prehabilitation paradigms have largely prioritized hypertrophy and aerobic conditioning [5,9]. However, the preoperative window in PDAC is frequently limited to 4–6 weeks in upfront surgery and biologically constrained by ongoing tumour-driven catabolism, even in the neoadjuvant setting [12]. Interventions aimed solely at increasing muscle mass may therefore be misaligned with the dominant drivers of vulnerability. By contrast, approaches that modulate inflammatory and metabolic stress, including immunonutrition, exercise-induced myokine signaling, ERAS-mediated metabolic optimization, or future cytokine-directed therapies, may more directly target the pathways implicated in PDAC-associated sarcopenia [12,13,40].

Experience from other gastrointestinal and thoracic malignancies provides proof of principle. Multimodal prehabilitation strategies that incorporate anti-inflammatory nutrition and metabolic conditioning have demonstrated improvements in immune recovery and treatment tolerance in patients with colorectal cancer [43]. More directly, pharmacologic targeting of cachexia-associated pathways, including activin/myostatin blockade and IL-6 pathway inhibition, has shown measurable biological effects in advanced malignancy settings by mitigating muscle loss, even when survival benefits have not been demonstrated in human studies [25,26,27,28,44]. Although such strategies have not yet been evaluated in resectable PDAC, they illustrate that upstream modulation of immunometabolic signaling may be biologically feasible.

For surgeons, this evolving understanding necessitates reconsideration of what constitutes meaningful perioperative success. Operative mortality and short-term morbidity remain important, but they may represent only an early checkpoint in a longer therapeutic journey. In PDAC, the ultimate objective of resection is not merely survival of the operation, but recovery sufficient to receive systemic therapy and sustain antitumor immunity. If sarcopenia reflects impaired capacity to achieve this transition, then prehabilitation should be designed not merely to increase muscle mass, but to optimize systemic resilience from an immunometabolic perspective.

Future perioperative trials in PDAC should therefore prioritize endpoints that reflect this broader objective, including chemotherapy completion rates, immune recovery metrics, and recurrence-free survival, rather than relying solely on short-term complication rates. Integrative trial designs that combine nutritional, exercise, and immunometabolic interventions may be particularly valuable in clarifying causality between pathway modulation and oncologic outcomes.

## 9. Conclusions

Sarcopenia in pancreatic ductal adenocarcinoma has long been interpreted as a surrogate for frailty or diminished physical reserve. However, the convergence of clinical outcome data, translational immune profiling, and mechanistic cachexia research suggests that this phenotype may reflect a more profound disruption of host immunometabolic homeostasis. The consistent association between sarcopenia, impaired tolerance of systemic therapy, and inferior survival, coupled with its inconsistent relationship to immediate postoperative morbidity, challenges the traditional framing of sarcopenia as merely a surgical risk factor [1,2,19].

Repositioning sarcopenia as a marker of systemic vulnerability rather than isolated muscle depletion has implications that extend beyond body composition measurement. It reframes prehabilitation from an attempt to rapidly augment muscle mass to a broader effort aimed at preserving immune competence and metabolic resilience during the perioperative and adjuvant phases of care. While definitive interventional data in resectable PDAC remain limited, emerging insights into inflammatory, cytokine-mediated, and tumour–host crosstalk pathways provide a biologically coherent foundation for this shift [24,29,33]. Understanding sarcopenia through an immunometabolic lens offers a framework for future trials and a pathway toward redefining surgical success not simply as operative survival, but as sustained oncological recovery.

## Data Availability

No new data were created or analyzed in this study. Data sharing is not applicable to this article.

## Abbreviations

The following abbreviations are used in this manuscript:

PDAC	Pancreatic Ductal Adenocarcinoma
SMI	Skeletal Muscle Index
IL-6	Interleukin-6
STAT3	Signal Transducer and Activator of Transcription 3
TGF-β	Transforming Growth Factor Beta
SMAD	Mothers Against Decapentaplegic Homolog (usually just defined as SMAD signaling proteins)
NF-κB	Nuclear Factor Kappa B
MDSC	Myeloid-Derived Suppressor Cell
mTOR	Mechanistic Target of Rapamycin
SMD	Skeletal Muscle Radiodensity
AMG	Albumin-Myosteatosis Gauge
ERAS	Enhanced Recovery After Surgery
ACVR2B	Activin Receptor Type IIB
KLF10	Kruppel-like Factor 10
ROS	Reactive Oxygen Species
NOX2	NADPH Oxidase 2
